# Cytokine Storm in Coronavirus Disease 2019 and Adult-Onset Still’s Disease: Similarities and Differences

**DOI:** 10.3389/fimmu.2020.603389

**Published:** 2021-01-19

**Authors:** Jianfen Meng, Yuning Ma, Jinchao Jia, Mengyan Wang, Jialin Teng, Hui Shi, Honglei Liu, Yutong Su, Junna Ye, Yue Sun, Xiaobing Cheng, Huihui Chi, Tingting Liu, Dehao Zhu, Zhuochao Zhou, Liyan Wan, Zhihong Wang, Fan Wang, Xin Qiao, Xia Chen, Hao Zhang, Zihan Tang, Chengde Yang, Qiongyi Hu

**Affiliations:** ^1^Department of Rheumatology and Immunology, Ruijin Hospital, Shanghai Jiao Tong University School of Medicine, Shanghai, China; ^2^Department of Rheumatology and Immunology, The Fourth Affiliated Hospital of Nantong University, The First People’s Hospital of Yancheng, Yancheng, China

**Keywords:** coronavirus disease 2019, adult-onset Still’s disease, cytokine storm, hyperferritinemia, inflammation

## Abstract

The catastrophic outbreak of coronavirus disease 2019 (COVID-19) is currently a public emergency. Adult-onset Still’s disease (AOSD) is an autoinflammatory disease characterized by life-threatening complications. Systemic hyperinflammation and cytokine storm play a critical role in the pathogenesis of both COVID-19 and AOSD. We aimed to compare the similarities and differences focusing on ferritin and cytokine levels between severe COVID-19 and active AOSD. A literature search was performed using the databases PubMed, EMBASE, and Web of Science to collect the levels of cytokine including IL-1β, IL-6, IL-18, TNF-α, IL-10, and ferritin in severe COVID-19 patients. After extracting available data of indicators of interest, we acquired these statistics with a single-arm meta-analysis. Furthermore, a comparison was conducted between 52 patients with active AOSD in our center and severe COVID-19 patients from databases. The levels of IL-6 and IL-10 were higher in severe COVID-19 compared with those in active AOSD. There were no significant differences on the cytokine of IL-1β and TNF-α. Fold changes of IL-18 were defined as the mean expression level ratio of severe COVID-19 to healthy controls in the COVID-19 study and active AOSD to healthy controls in our study, individually. Although the fold change of IL-18 in patients with AOSD was significantly higher than patients with severe COVID-19 (fold change: 594.00 vs 2.17), there was no statistical comparability. In addition, the level of ferritin was higher in active AOSD in comparison with severe COVID-19. Our findings suggest that severe COVID-19 and active AOSD have differences in cytokine panel and ferritin level, indicating the pathogenic role of ferritin in overwhelming inflammation. And it paves the way to make efficacy therapeutic strategy targeting the hyperinflammatory process in COVID-19 according to AOSD management, especially in severe COVID-19.

## Introduction

Coronavirus disease 2019 (COVID-19), a novel virus-induced acute respiratory disease syndrome, has placed much pressure on healthcare systems with its high mortality rate (1). Intriguingly, COVID-19 demonstrates a heterogeneous course, from asymptomatic manifestations to severe respiratory involvement, multiorgan dysfunction, and even death. Accordingly, patients with COVID-19 were divided into two groups: asymptomatic or mild subset and severe subset (2). The common symptoms of COVID-19 were fever, dry cough, myalgia, and dyspnea.

A hallmark of COVID-19 is an excessive release of pro-inflammatory cytokines including interleukin-6 (IL-6), interleukin-1α (IL-1α), interleukin-1β (IL-1β), tumor necrosis factor-α (TNF-α), granulocyte-macrophage colony-stimulating factor (GM-CSF), and monocyte chemoattractant protein 1 (MCP1), which is an overwhelming inflammatory process called a cytokine storm. Compared with non-intensive care unit (ICU) patients with COVID-19, increased levels of cytokines, such as interleukin-2 (IL-2), interleukin-7 (IL-7), interleukin-10 (IL-10), and TNF-α, were observed in ICU patients with COVID-19, suggesting the potential pathogenetic role of exuberant cytokine in COVID-19 (3). Meanwhile, increasing evidence suggests that severe COVID-19 patients at higher risk of poor outcome have higher ferritin levels in serum (1297.6 ng/ml in non-survivors vs 614.0 ng/ml in survivors) (4), leading to a hypothesis that ferritin may not only act as a bystander of acute phase response but also play a pivotal role in inflammation milieu in COVID-19. Ferritin, an inflammatory protein regulated by pro-inflammatory cytokines, may further enhance pro-inflammatory process in inflammatory setting of COVID-19, contributing to the development of cytokine storm in COVID-19 (5, 6). It has spurred comparisons with other severe diseases that are associated with increased cytokines and ferritin.

Adult-onset Still’s disease (AOSD), also known as a hyperferritinemic syndrome, is a multi-system involved autoinflammatory disease. Also triggered by viral infections, AOSD is characterized by high spiking fever, evanescent skin rash, sore throat, polyarthralgia, and even life-threatening complications, including fulminant hepatitis and macrophage activation syndrome (MAS) (7, 8). Similarly, excessive and uncontrolled production of cytokines, including IL-1β, IL-6, interleukin-18 (IL-18), and TNF-α, has been recognized as a cornerstone in AOSD pathogenesis (9). It is still matter of debate about the differences of cytokine panel in COVID-19 and AOSD, the two typical conditions of cytokine storm.

Furthermore, due to the similarity of clinical presentations and high levels of ferritin, AOSD, MAS, catastrophic antiphospholipid syndrome, and septic shock are included under the umbrella of hyperferritinemic syndrome (10). In fact, AOSD patients have a largely over-abundant level of ferritin. Due to hyperferritinemia in severe COVID-19, it is hypothesized that severe COVID-19 is a new member of hyperferritinemic syndrome (6, 10). Additionally, severe COIVD-19 and AOSD might share a common pathogenesis of cytokine storm. On the basis of shared features of cytokine storm and hyperferritinemia in AOSD and severe COVID-19, it would be extremely interesting to evaluate the similarities and differences to better understanding of the pathogenesis of cytokine storm. Consequently, we performed our study to compare the similarities and differences focusing on the ferritin and cytokine level between severe COVID-19 and AOSD.

## Methods

### Subjects and Cytokine Assessment

A total of 52 active AOSD patients were enrolled from Department of Rheumatology and Immunology, Ruijin Hospital, Shanghai Jiao Tong University. All patients with AOSD were diagnosed according to Yamaguchi’s criteria after exclusion of infectious, neoplastic, and autoimmune diseases (11). The systemic disease activity of each AOSD patient was assessed using a modified Pouchot’s score (12). Patients with active AOSD were defined by the presence of fever and/or skin rash and/or inflammatory arthralgia/arthritis and/or sore throat (13). Forty-two age- and sex-matched health volunteers were recruited as healthy controls. Serum levels of IL-1β, IL-6, IL-18, TNF-α, and IL-10 in AOSD patients were detected using the Meso Scale Discovery electrochemiluminescence assay (MSD, Rockville, MD, USA) as described previously (13). Ferritin level in serum was quantified using commercial ELISA reagent kit following the manufacturer’s instructions.

### Search Strategy and Selection Criteria

We searched PubMed, EMBASE, and Web of Science databases to identify investigations reporting COVID-19 with cytokine levels using the following search terms: (“coronavirus” OR “COVID-19” OR “SARS-CoV-2” OR “novel coronavirus” OR “coronavirus disease 2019” OR “Wuhan virus” OR “China coronavirus” OR “SARS-coronavirus-2) AND (“cytokine” OR “interleukin”). The search process was restricted to English-language articles involving humans dated from December 2019 to July 18, 2020. The search strategy conformed to the Preferred Reporting items for Systematic Reviews and Meta-Analyses (PRISMA) statement (**Table S1**). Full-text of research articles was eligible if they reported exact data of cytokine level in severe COVID-19. In our study, patients with COVID-19 receiving treatment in intensive care unit or non-survivor or SpO2<90% or required mechanical ventilation or complicated with ARDS were considered severe. In some cases, hospitalized patients were also regarded as severe, compared with ambulatory patients. Review articles, editorials, comments, case reports, letters, and researches on pediatrics, pregnancy, and obstetrics were excluded. Meanwhile, articles in the MedRxiv or unaccepted articles were also excluded. During the search process, a PICOs format was used (**Table S2**). Owing to the characteristics of single-arm meta analysis in our study, the comparison and outcome were not required during the PICOs process.

### Risk of Bias Assessment

To critically assess the risk of bias for the included studies, the following criteria were applied in our study, which was similar to the assessment by Bao et al. (14). The criteria included a clear purpose of the study, including continuous patients, the definition of severe COVID-19, sufficient data of interest, the end-point adapted to the research goal, and a fair assessment of the end-point. The item not reported was scored 0, reported but insufficient was 1, reported and sufficient was 2. The global score of this scale was 14. Studies with a score ≥ 10 were considered high quality, and then were selected for further analysis; otherwise, they were excluded. Additionally, we drafted funnel-plots for indicators to assess publication bias if there were at least 10 included studies. When asymmetry of the funnel plots was present, the trim and fill methods were further applied to adjust publication bias.

### Data Extraction and Analysis

Two independent reviewers (JM and YM) performed the literature search and assessed each article for inclusion and exclusion. Discrepancies were resolved by a third investigator (QH). The results of initial search were first screened by title and abstract. Then, the full-text of relevant articles was reviewed for inclusion and exclusion criteria. We extracted the following data from selected articles: author, publication date, journal, number of enrolled COVID-19 patients, country, study type, age, gender, ferritin, exact data of cytokine level. Data of cytokine and ferritin presented as median (interquartile, IQR) or median (Min-Max) were estimated to mean (standard deviation, SD) by Hozo group’s method (15). Additionally, we utilized *I^2^* statistic to assess the heterogeneity of the included studies. When *I^2^* > 75%, a random-effects model was used to minimize inter-study heterogeneity. The Stata version 16.0 was used to perform overall mean analysis. *P*-values less than 0.05 were considered statistically significant.

## Results

### Characteristics of Included Studies

**Figure 1:**
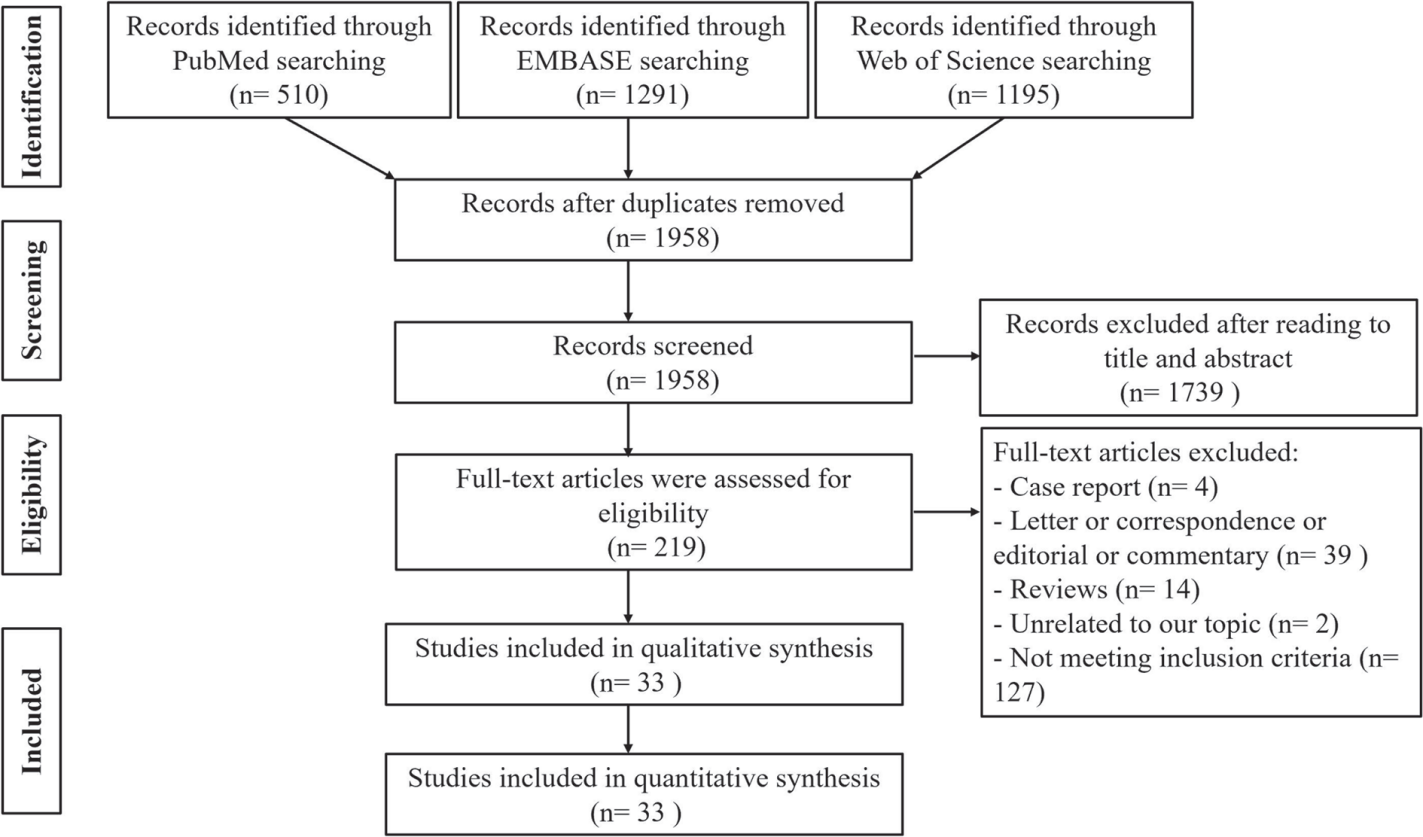
The process of the literature search and selection.

The steps of literature search are presented in **Figure 1**. A total of 2,996 publications were retrieved from PubMed, EMBASE, and Web of Science databases, 1,038 records of which were excluded due to duplication. After screening the remaining 1,958 publications according to title and abstraction, 219 full-text articles were further accessed for eligibility. Finally, we obtained the cytokine results of 33 articles describing 1,992 patients with COVID-19 (16–48).

Among these articles, 9 articles described the results of IL-1β, 31 articles demonstrated the results of IL-6, 16 studies presented the levels of IL-10, 17 reports showed the levels of TNF-α, and only one article depicted data of IL-18. Meanwhile, we obtained data of ferritin from 12 articles among 33 selected articles. The characteristics, cytokine profiles, and ferritin level of 33 included studies were summarized in **Table 1**.

**Table 1:** Summary characteristics of 33 studies that demonstrated the cytokine level in patients with COVID-19.

Author	Journal	Year	Country	M, F	Age, years	IL-1β (pg/ml), measurement	IL-6 (pg/ml), measurement	IL-10 (pg/ml), measurement	IL-18(pg/ml), measurement	TNF-α (pg/ml), measurement	Ferritin (ng/ml)
Zhu et al. (16)	*Int J Infect Dis*	2020	China	9, 7	57.5 ± 11.7	None	26.5 ± 43.3, NA	6.9 ± 6.3, NA	None	1.5 ± 0.3, NA	None
Zheng et al. (17)	*J Zhejiang Univ-Sci B*	2020	China	11, 23	66.0 ± 14.0	None	50.6 ± 44.1, ELISA	6.7 ± 3.6, ELISA	None	None	None
Zheng et al. (18)	*Int J Infect Dis*	2020	China	21 (total)	60.7 ± 49.3	None	168.9 ± 346.0, NA	None	None	None	None
Zhang et al. (19)	* J Diabetes Complications*	2020	China	18, 9	70.3 ± 18.0	None	51.8 ± 62.3, NA	None	None	None	None
Zhang et al. (20)	* J Clin Virol*	2020	China	14, 4	63.3 ± 26.5	None	43.4± 61.1, NA	5.9 ± 2.7, NA	None	2.1 ± 0.4, NA	None
Zhang et al. (21)	*Clinica chimica acta*	2020	China	55, 28	65.3 ± 12.1	5.0 ± 0, NA	102.0 ± 141.5, NA	10.4 ± 8.3, FC	None	12.6 ± 9.7, NA	None
Zhang et al. (22)	*PloS one*	2020	China	54, 28	72.5 ± 11.3	None	208.7 ± 118.3, CL	None	None	None	None
Yan et al. (23)	*BMJ Open Diabetes Res Care*.	2020	China	76, 32	70.0 ± 12.0	None	73.6 ± 85.4, NA	None	None	13.5 ± 8.9, NA (n=39)	1823.3 ± 1237.6
Xu et al. (24)	*Journal of Infection*	2020	China	11, 17	72.8 ± 7.2	6.6 ± 3.9, NA	36.1 ± 38.5, NA	13.9 ± 13.3, NA	None	12.5 ± 3.5, NA	None
Wu et al. (25)	*mSphere*	2020	China	27, 12	62.7 ± 13.9	None	24.4 ± 33.3, FC	6.4 ± 5.6, FC	None	0.1 ± 0.2, FC	None
Wang et al. (26)	*Clin Infect Dis*	2020	China	7, 7	69.8 ± 12.4	None	82.6 ± 117.0, NA	7.6 ± 6.7, FC	None	2.1 ± 0.4, NA (n=7)	None
Wang et al. (27)	*J Infect Dis*	2020	Italy	10, 1	59.7 ± 19.5	None	44.4 ± 66.1, FC	6.6 ± 6.2, FC	None	1.7 ± 0.7, FC	None
Wang et al. (28)	*Endocrine Practice*	2020	China	10,4	71.4 ± 7.9	None	129.0 ± 111.04, NA	13.1 ± 10.5, CL	None	15.2 ± 9.7, NA	1716.0 ± 860.0
Wan et al. (29)	*Br J Haematol*	2020	China	21 (total)	61.3 ± 15.6	None	37.8 ± 7.8, FC	4.6 ± 0.4, NA	None	2.9 ± 0.4, FC	None
Vultaggio et al. (30)	*J Allergy Clin Immunol Pract*	2020	Italy	46, 17	66.0 ± 15.0	None	53.6 ± 63.8, ELISA	None	None	None	None
Tian et al. (31)	*Lancet Oncology*	2020	China	81, 67	63.7 ± 8.2	5.0 ± 0, CL	24.2 ± 32.6, CL	6.8 ± 4.1, NA	None	9.5 ± 4.7, CL (n=68)	1125.1 ± 1404.7
Sun et al. (32)	*J Am Geriatr Soc*	2020	China	82, 39	72.00 ± 9.00	None	91.1 ± 95.8, NA	None	None	None	1680.0 ± 1117.9
Song, et al. (33)	*Nat Commun*	2020	China	8, 4	58.3 ± 37.9	1.1 ± 1.1, FC	15.9 ± 18.5, FC	3.8 ± 2.0, FC	53.2 ± 26.5, FC	3.9 ± 2.6, FC	596.43 ± 350.5
Luca et al. (34)	*J Med Virol*	2020	Italy	4, 2	68.8 ± 9.4	None	276.0 ± 568.2, CL	None	None	None	None
Austin R et al. (35)	*J Autoimmun*	2020	USA	27, 8	63.3 ± 16.2	None	48.3 ± 51.4, NA	None	None	None	2085.0± 1289.3, None
Mikamiet al. (36)	*J Gen Intern Med*	2020	USA	323, 483	75.3 ± 14.9	0.7 ± 0.6, NA	178.4 ± 166.9, NA	None	None	28.0 ± 13.8, NA (n=806)	1185.3 ± 1302.6
Liu et al. (37)	*Viral Immunology*	2020	China	30 (total)	/	7.2 ± 5.1, NA	40.2 ± 66.8, NA	7.9 ± 4.8, NA	None	None	None
Li et al. (38)	*JCI insight*	2020	China	26, 17	40.3 ± 18.4	11.4 ± 9.4, CL	29.2± 20.8, CL	None	None	7.6 ± 1.5, CL	835.7 ± 753.2
Huang et al. (39)	*J Med Virol*	2020	China	5, 5	38.6 ± 11.4	12.0 ± 17.9, NA	23.2 ± 7.6, NA	32.2 ± 38.9, NA	None	25.6 ± 29.7, NA	None
Tobias et al. (40)	*J Allergy Clin Immunol*	2020	UK	29, 3	63.7 ± 27.9	None	179.9 ± 322.7, NA	None	None	None	1735.3 ± 2591.9
Gao et al. (41)	*Journal of Medical Virology*	2020	China	6, 9	45.2 ± 7.7	None	39.4 ± 29.6, CL	None	None	None	None
Gan et al. (42)	*Front Public Health*	2020	China	28, 11	71.3 ± 7.7	None	102.4 ± 96.5, NA	6.3 ± 7.7, NA	None	12.3 ± 5.6, NA	None
Michael et al. (43)	*Dtsch Arztebl Int*	2020	Germany	15, 9	63.3 ± 9.5	None	168.0 ± 229.3, NA	None	None	None	None
Erol et al. (44)	*Transpl Infect Dis*	2020	Turkey	3, 4	49.3 ± 14.8	None	None	None	None	None	934.6 ± 1508.2
Chen et al. (45)	*J Clin Lab Anal*	2020	China	15, 5	69.8 ± 9.9	5.0 ± 0, NA	81.6 ± 91.9, NA	11.2 ± 8.6, NA	None	13.0 ± 10.5, NA	1503.3 ± 905.6
Chen et al. (46)	*JCI*	2020	China	10, 1	61.2 ± 8.1	None	None	None	None	None	1686.3 ± 518.6
Viviana et al. (47)	*Monaldi Arch Chest Dis*	2020	Turkey	8, 2	73.0 ± 7.4	None	58.5 ± 48.6, NA	None	None	None	None
Burian et al. (48)	*J. Clin. Med*	2020	Germany	22, 6	64.9 ± 16.6	None	103.9 ± 43.6, NA	None	None	None	None

COVID-19, coronavirus disease 2019; M, male; F, female; IL, interleukin; TNF-α, tumor necrosis factor-α; FC, flow cytometry; CL, chemiluminescence; ELISA, enzyme-linked immunosorbent assay. NA, not available. Data are presented as mean ± standard deviation or n.

### The Differences of Clinical Manifestations and Laboratory Parameters Between COVID-19 and Active AOSD

The clinical characteristics of COVID-19 came from China, including 1,099 patients (1). Patients with active AOSD were younger than COVID-19 (38.8 ± 15.2 vs 46.7 ± 17.1 years, *P* = 0.0011), with high female predominance. Fever was the common manifestation in COVID-19 and active AOSD (88.7% vs 90.4%, *P* = 0.7095), whereas AOSD patients were characterized by sore throat (46.2% vs 13.9%, *P* < 0.0001), skin rash (76.9% vs 0.2%, *P* < 0.0001), arthralgia or myalgia (71.2% vs 14.9%, *P* < 0.0001), and hepatosplenomegaly or lymphadenopathy (73% vs 0.2%, *P* < 0.0001). The number of lung involvement assessed by chest CT was increased in patients with COVID-19 when comparing with AOSD (86.2% vs 17.3%, *P* < 0.0001). The occurrence of digestive symptoms, acute respiratory distress syndrome, coagulopathy, acute kidney injury, and death was higher in COVID-19, while no statistical difference was found between COVID-19 and AOSD. Regarding laboratory parameters, white blood cell (12.2 ± 6.2 vs 4.7 ± 1.9 10^9^/L, *P* < 0.0001), platelets (256.5 ± 92.6 vs 169.0 ± 55.7 10^9^/L, *P* < 0.0001), and lymphocyte (1.3 ± 0.75 10^9^/L vs 1.0 ± 0.4 10^9^/L, *P* < 0.0001) in AOSD were higher than those in COVID-19. Furthermore, the proportions of abnormal liver function tests (51.9% vs 22.2%, *P* < 0.0001) and elevated C-reactive protein level (90.2% vs 60.7%, *P* < 0.0001) were significantly higher in AOSD patients than in patients with COVID-19. The detailed comparison of clinical manifestations and laboratory parameters between COVID-19 and active AOSD is presented in **Table 2**.

**Table 2:** Comparison of clinical manifestations and laboratory parameters between COVID-19 and active AOSD.

Variables	COVID-19 (n=1,099)	Active AOSD (n= 52)	*P* value
Age, years	46.7 ± 17.1	38.8 ± 15.2	**0.0011**
Female, %	459 (41.9)	42 (76.9)	**< 0.0001**
Infection as trigger	SARS-CoV-2	Virus as probable trigger	/
Fever, %	975 (88.7)	47 (90.4)	0.7095
Sore throat, %	153 (13.9)	40 (46.2)	**< 0.0001**
Skin rash, %	2 (0.2)	37 (76.9)	**< 0.0001**
Arthralgia or myalgia, %	164 (14.9)	37 (71.2)	**< 0.0001**
Arthritis, %	Not available	14 (26.9)	/
Hepatosplenomegaly or lymphadenopathy, %	2 (0.2)	38 (73)	**< 0.0001**
Nausea or vomiting, %	55 (5.0)	0 (0)	0.1709
Diarrhea, %	42 (3.8)	0 (0)	0.2551
Abnormalities on chest CT— no./total no. (%)	840/975 (86.2)	9/52 (17.3)	**< 0.0001**
Acute respiratory distress syndrome, %	37 (3.4)	0 (0)	0.4073
Coagulopathy, %	1 (0.1)	0 (0)	> 0.9999
Acute kidney injury, %	6 (0.5)	0 (0)	> 0.9999
Death, %	15 (1.4)	0 (0)	> 0.9999
White blood cell, (*10^9^/L)	4.7 ± 1.9	12.2 ± 6.2	**< 0.0001**
Lymphocyte count, (*10^9^/L)	1.0 ± 0.4	1.3 ± 0.75	**< 0.0001**
Neutrophil count, (*10^9^/L)	Not available	9.7 ± 5.9	/
Hemoglobin, (g/L)	133.7 ± 21.5	106.1 ± 20.5	**< 0.0001**
Platelets, (*10^9^/L)	169.0 ± 55.7	256.5 ± 92.6	**< 0.0001**
Abnormal liver function— no./total no. (%)	168/757 (22.2)	26/52 (51.9)	**< 0.0001**
C-reactive protein (≥10 mg/l)— no./total no. (%)	481/793 (60.7)	46/51 (90.2)	**< 0.0001**
Modified Pouchot score, median (IQR)	/	5.0 (3.0-5.7)	**/**

COVID-19, coronavirus disease 2019; AOSD, adult-onset Still’s disease. IQR (inter-quartile ranges). Data are presented as mean ± standard deviation or n (%).

P < 0.05 is shown in bold type.

### Comparison of the Cytokine Profile and Ferritin Between Severe COVID-19 and Active AOSD

The comprehensive collation of the expression of cytokine profile and ferritin data is shown in **Table 3**. Compared with active AOSD, patients with severe COVID-19 with higher male predominance were older than active AOSD, which was parallel to the results of **Table 2**. The levels of IL-6 and IL-10 were higher in severe COVID-19 compared with that in active AOSD. The levels of IL-1β tended to be elevated in severe COVID-19, while there was no significant difference between severe COVID-19 and active AOSD. Also, the level of TNF-α was comparable between these two diseases. Only one investigation reported the exact raw data on IL-18 with healthy control and patients with COVID-19. Fold changes of IL-18 were defined as the mean expression level ratio of severe COVID-19 and AOSD to healthy controls. Noteworthy, the fold change of IL-18 in patients with active AOSD was approximately 594, which was much higher than that in severe COVID-19 (Fold change 2.17), without statistical comparability. In addition, the level of ferritin was significantly higher in active AOSD in comparison with severe COVID-19 (1905.0 ± 1509.00 vs 1399.0 ± 1294.5ng/ml, *P*=0.0413). In addition, the forest plots of age, IL-1β, IL-6, IL-10, TNF-α, and ferritin were presented in **Figure S1-6**.

**Table 3:** Comparative results of cytokine and ferritin level between severe COVID-19 and active AOSD using meta-analysis.

Variables	Included studies	Patients with severe COVID-19, n	Patients with active AOSD, n	*P* value
				COVID-19 vs AOSD
Age, years	32	64.07 ± 10.36, 1946	38.8 ± 15.2, 52	***P* < 0.0001**
Male (%)	32	1040 (54.6)	10 (19.2)	***P* < 0.0001**
IL-1β, pg/ml	9	5.38 ± 18.8, 943	0.47 ± 1.10, 47	0.0755
IL-6, pg/ml	31	68.37 ± 53.15, 1829	15.98 ± 20.75, 52	***P* < 0.0001**
IL-10, pg/ml	16	7.27± 2.98, 447	3.99 ± 5.07, 30	**0.0043**
IL-18	1	Fold changes to HC: 2.17	Fold changes to HC: 594	**/**
TNF-α, pg/ml	17	8.23 ± 5.99, 1272	8.27 ± 6.20, 52	0.9710
Ferritin, ng/ml	12	1399.0 ± 1294.5, 1183	1905.00 ± 1509.00, 45	**0.0413**

COVID-19, coronavirus disease 2019; AOSD, adult-onset Still’s disease; IL, interleukin; TNF, tumor necrosis factor. Data are presented as mean ± standard deviation or n (%).

P < 0.05 is shown in bold type.

### Quality Assessment

Judging the assessment criteria, all 33 included studies were classified as high quality (**Table S3**). As shown in Figure S7, distribution of the funnel plot was symmetric in ferritin, indicating no evidence of publication bias. However, it was asymmetric in the funnel plots of age, IL-6, IL-10, TNF-α (**Figure S8**). Next, the trim and fill methods were applied to adjust publication bias. After adjustment, the distribution of the funnel plots in age (**Figure S8**) was more symmetric than before, indicating the results of age were relatively stable. However, the asymmetry of funnel plots of IL-6, IL-10, TNF-α still exited (**Figure S8**), which suggested the probable presence of the publication bias.

## Discussion

COVID-19, caused by a new strain of β-coronavirus, SARS-CoV-2, is emerging as a huge threat to human health (49). SARS-CoV-2 infection invokes a hyperinflammatory state driven by multiple immune cells and mediators including IL-6, IL-1α, IL-1β, and TNF-α. Broadly speaking, cytokine storm denotes a hyperactive inflammatory response characterized by systemic inflammation, multi-organ dysfunction, elevated IL-1β, IL-18, IL-6, interferon-γ (IFN-γ), TNF-α, ferritin and other mediators, which are injurious to host cells. The imitating factors leading to cytokine storm are heterogeneous, including infections, tumor, and rheumatologic origins (50). Elevated plasma concentration of inflammatory mediators was observed in patients with COVID-19, including IL-6, IL-10, TNF-α, and other inflammatory cytokines, as well as ferritin and C-reactive protein (3, 46). Cytokine storms play a critical role in the pathogenesis of AOSD, an inflammatory disease with high serum ferritin and neutrophil activation. Severe COVID-19 patients at higher risk of poor outcome also have raised levels of ferritin. Accumulating evidences have strongly indicated the pathogenetic role of ferritin in systemic hyperinflammation, more than a bystander. For these reasons, we further investigated the similarities and differences focusing on the cytokine panel and ferritin.

In our study, cytokines including IL-1β, IL-6, IL-10, and TNF-α were elevated in severe COVID-19 and active AOSD, compared with healthy controls, indicating the potential contribution of cytokine storm in pathogenesis of COVID-19 and AOSD. As a whole, the levels of IL-6 and IL-10 were significantly higher in severe COVID-19 from our results, suggesting a more serious cytokine storm underlying the pathogenesis of COVID-19. Consistent with the aforementioned results, the mortality of overall patients with COVID-19 was higher than active AOSD despite no statistical significance. The reported mortality of severe COVID-19 over 10% further indicated the contribution of cytokine storm to severe COVID (51). Previous reports showed that COVID-19 patients with worse prognosis had higher levels of IL-6 (3, 26, 52). Furthermore, targeting IL-6 signaling, i.e., tocilizumab, an IL-6 receptor antagonist, showed promising and encouraging results in the treatment of COVID-19 (53). Similarly, tocilizumab was effective on systemic manifestations of AOSD in a double-blinded randomized clinical trial (54). Based on the results from this trial, tocilizumab is approved for the treatment of AOSD. In a word, IL-6 is increased in COVID-19 and AOSD and the therapy targeting it is effective, indicating a potential and potent role in the pathogenesis of cytokine storm. IL-10, an anti-inflammatory cytokine, which was increased in severe COVID-19 patients (55), was correlated with disease activity of AOSD (56). The compensatory role of anti-inflammatory cytokine might be a shared phenomenon in the pathogenesis of inflammatory disease characterized by a cytokine storm. Although IL-18 was elevated in COVID-19 (33), the levels of IL-18 in AOSD of our study were significantly higher than in severe COVID-19 patients, indicating the potential distinguishing feature of cytokine profiles between COVID-19 and AOSD. Nearly two decades ago, IL-18 was first identified as a diagnostic marker and was an indicator of disease activity in AOSD (57). Blocking IL-18 with recombinant human IL-18 BP (tadekinig alfa) appears to have treatment efficacy in AOSD (58). As we know, IL-18 is derived from the inflammasome activation, which might be a contributor of the pathogenesis of AOSD (59). Due to lack of researches on inflammasome and COVID-19 currently, we speculate that inflammasome activation might play a minimal role in COVID-19 pathogenesis. IFN-γ, an important cytokine of cytokine storm, were not assessed in our study as serum samples of some AOSD patients in our study are not available. In COVID-19, enhanced levels of IFN-γ was correlated with the viral load and was considered a marker of poor outcome (3, 60). Similarly, serum level of IFN-γ and IFN-γ expression in natural killer cell were enhanced in acute AOSD and reduced in remission AOSD (12, 61). IFN-γ was released by hematopoietic cells during viral infection, where IFN-γ might boost the amplification of inflammation (62). Consequently, enhanced expression of IFN-γ was found in SARS-CoV-2 infection and AOSD triggered probably by virus. However, whether the levels of IFN-γ are comparable need further investigation. With regards to ferritin, several investigations have confirmed that ferritin was elevated in patients with COVID-19 and was correlated with disease severity (63, 64). Due to the similarities in clinical manifestations and high level of ferritin in serum, severe COVID-19 was regarded as a fifth member of hyperferritinemic syndromes (65). Ferritin, the common denominator of COVID-19 and AOSD, comprises 24 subunits of two types, heavy and light subunits. Beyond its iron storage role, ferritin functions in a pathogenic role in inflammation. Under inflammatory conditions, such as COVID-19 and AOSD, ferritin is upregulated in response to IL-1β, IL-6, and IFN-γ. Furthermore, ferritin further stimulates inflammatory pathways to amply the inflammatory process (5, 66–68). Interestingly, the expression of ferritin was dramatically increased in AOSD when comparing with COVID-19, which was consistent with the analysis from Colafrancesco et al. (65). In vitro experiments showed that ferritin might be secreted by hepatocytes and macrophages (69). A previous study demonstrated that ferritin levels are correlated with disease activity and macrophage activation in AOSD (70). Accordingly, we hypothesize that the contribution of macrophage activation is more crucial to AOSD compared with COVID-19, resulting in the higher levels of ferritin in AOSD.

Besides denominators of hyperferritinemia and cytokine storm in COVID-19 and AOSD, there are also shared clinical manifestations. Fever, as a result of cytokine storm, is not surprisingly the most prominent common feature of COVID-19 and AOSD. Meanwhile, sore throat, arthralgia, or arthritis are relatively common in these two diseases. From our comparative results, digestive symptoms, such as nausea or vomiting, and diarrhea are comparable. AOSD, unlike COVID-19, is frequently triggered by viral infections with uncertain origins, while SARS-CoV-2 is the leading cause of COVID-19. The cardinal target organ of COVID-19 is lung, causing an acute respiratory distress syndrome. Recently, Ruscitti et al. conducted a study to compare the difference of clinical manifestation, laboratory tests, and radiological imaging in patients with lung involvement between severe COVID-19 and MAS secondary to AOSD (71). More patients in the COVID-19 group presented with ground-glass opacities (GGOs) compared with those in the group of MAS secondary to AOSD. And higher proportions of apical, basal, bilateral, and peripheral distributions of GGOs were observed in COVID-19 patients than in MAS. Consistent with this feature, SARS-CoV-2 is transmitted by droplets that come into the respiratory system, which widely express the angiotensin-converting enzyme-related carboxypeptidase as receptors of SARS-CoV-2. This may be the underlying reason why more COVID-19 patients suffered from lung involvement. The clinical manifestations, including arthralgia or arthritis, skin rashes, hepatomegaly, splenomegaly as distinctive clinical manifestations of AOSD haven’t been reported in COVID-19 so far. Furthermore, increased count of leukocyte and neutrophil is remarkable in AOSD, which is opposite to COVID-19 in which leukopenia are common. Besides these features mentioned above, diffused intravascular coagulopathy (DIC) is a rare but serious complication in patients with COVID-19 and AOSD. Regarding treatment, glucocorticoids, which are not recommended in COVID-19 unless severe patients, have been considered as the first-line therapy of AOSD patients.

Our study has several limitations. First, relatively few studies with exact data of IL-1β and IL-18 were included in this analysis, resulting in the difficulty of interpretation of the results. And the *I^2^* statistics of our meta-analysis were high, indicating large heterogeneity, probably secondary to the difference of measurement in our selected studies. Therefore, the conclusion of our study needs to be verified by unified measurement of cytokines in more COVID-19 patients and healthy controls. Second, in the majority of patients with COVID-19 in our meta-analysis, the article described clinical manifestation and our AOSD cohort came from China, suggesting an underlying ethnic bias. Additional data from all over the world would provide a more comprehensive picture to better understand the similarities and differences between COVID-19 and AOSD. Whether the conclusion in our study is consistent with other studies consisting of more ethnicities remains to be investigated. Third, IL-18 binding protein (IL-18BP), a naturally IL-18 inhibitor in the circulation, can distinguish free bioactive IL-18 from total IL-18. The level of IL-18BP is not usually detected in COVID-19, thus a comparison of IL-18 binding protein (IL-18BP) level is not performed in our study (72). Last, potential publication bias and availability of data are mainly common limitations of all meta-analyses, leading us to explain the results cautiously.

## Conclusions

In conclusion, our findings suggest that both COVID-19 and AOSD have elevated level of ferritin and cytokines, including IL-1β, IL-6, IL-10, IL-18, and TNF-α, indicating systemic inflammation in the pathogenesis of COVID-19 and AOSD. Importantly, all triggered by virus, COVID-19 and AOSD have differences in cytokine panel and ferritin level, indicating the diverse pathogenic role of cytokines and ferritin in overwhelming inflammation. And it paves the way to make efficacy therapeutic strategy targeting the hyperinflammatory process in COVID-19 according to AOSD management, especially severe COVID-19.

## Data Availability Statement

The original contributions presented in the study are included in the article/**Supplementary Material**. Further inquiries can be directed to the corresponding authors.

## Ethics Statement

Information on demographic and clinical data were obtained under a protocol approved by the Institutional Research Ethics Committee of Ruijin Hospital (ID: 2016-62), Shanghai, China. The patients/participants provided their written informed consent to participate in this study.

## Author Contributions

Study conception or design: QH, CY, JM. Acquisition of the data: JM, YM, JJ, MW, JT, HS, HL, YTS, JY, YS, XBC, HC, TL, DZ, ZZ, LW, ZW, FW, XQ, XC, HZ, and ZT. Analysis and interpretation of the data: JM, YM, JJ, MW, and QH. Drafting and revising the article: QH, CY, and JM. All authors contributed to the article and approved the submitted version.

## Funding

This work was supported by the National Natural Science Foundation of China (82001704), Shanghai Sailing Program (20YF1427100), Shanghai Pujiang Young Rheumatologists Training program (SPROG201901), and Shanghai Science and Technology Innovation Action (20Y11911500).

## Conflict of Interest

The authors declare that the research was conducted in the absence of any commercial or financial relationships that could be construed as a potential conflict of interest.

## Abbreviations

COVID-19, Coronavirus disease 2019; IL-6, interleukin-6; IL-1α, interleukin-1α; IL-1β, interleukin-1β; TNFα, tumor necrosis factor-α; GM-CSF, granulocyte-macrophage colony-stimulating factor; MCP1, monocyte chemoattractant protein 1; ICU, non-intensive care unit, IL-2, interleukin-2; IL-7, interleukin-7; IL-10, interleukin-10; IL-18, interleukin-18; AOSD, Adultonset Still’s disease; MAS, macrophage activation syndrome; PRISMA, Preferred Reporting items for Systematic Reviews and Meta-Analyses statement; IQR, interquartile; SD, standard deviation; IFN-γ, interferon-γ; GGOs, ground-glass opacities, DIC, diffused intravascular coagulopathy.
