# Supplementary material for: Cytokine Storm in Coronavirus Disease 2019 and Adult-Onset Still’s Disease: Similarities and Differences
Source: Front Immunol. 2021 Jan 19;11:603389. doi: 10.3389/fimmu.2020.603389 (PMC7856388; doi:10.3389/fimmu.2020.603389)
Supplement: Supplementary file 1 [file DataSheet_1.docx]

**Cytokine storm in coronavirus disease 2019 (COVID-19) and adult-onset Still's disease: similarities and differences**

Jianfen Meng^1, 2,^†, Yuning Ma^1,^†, Jinchao Jia^1,^†, Mengyan Wang^1,^ †, Jialin Teng^1^, Hui Shi^1^, Honglei Liu^1^, Yutong Su^1^, Junna Ye^1^, Yue Sun^1^, Xiaobing Cheng^1^, Huihui Chi^1^, Tingting Liu^1^, Dehao Zhu^1^, Zhuochao Zhou^1^, Liyan Wan^1^, Zhihong Wang^1^, Fan Wang^1^, Xin Qiao^1^, Xia Chen^1^, Hao Zhang^1^, Zihan Tang^1^, Chengde Yang^1, *^, Qiongyi Hu^1,*^

1. Department of Rheumatology and Immunology, Ruijin Hospital, Shanghai Jiao Tong University School of Medicine, Shanghai, China.
2. Department of Rheumatology and Immunology, The First People's Hospital of Yancheng, The Forth Affiliated Hospital of Nantong University, Yancheng 224006, China.

†These authors contributed equally to this work.

* **Correspondence to:** Qiongyi Hu or Chengde Yang

Qiongyi Hu

1.Department of Rheumatology and Immunology, Ruijin Hospital, Shanghai Jiao Tong University School of Medicine, No. 197 Ruijin Second Road, Shanghai 200025, China

Tel.: (86)-21-64370045ext665130; Fax: (86)-21-54109718

Email: huqiongyi131@163.com; Chengde Yang, Email: [yangchengde@sina.com](mailto:yangchengde@sina.com)

| **Table S1 PRISMA 2009 Checklist** | | | |
| --- | --- | --- | --- |
| **Section/topic** | **#** | **Checklist item** | **Reported on page #** |
| **TITLE** | | |  |
| Title | 1 | Identify the report as a systematic review, meta-analysis, or both. | - |
| **ABSTRACT** | | |  |
| Structured summary | 2 | Provide a structured summary including, as applicable: background; objectives; data sources; study eligibility criteria, participants, and interventions; study appraisal and synthesis methods; results; limitations; conclusions and implications of key findings; systematic review registration number. | 2 |
| **INTRODUCTION** | | |  |
| Rationale | 3 | Describe the rationale for the review in the context of what is already known. | 3-4 |
| Objectives | 4 | Provide an explicit statement of questions being addressed with reference to participants, interventions, comparisons, outcomes, and study design (PICOS). | - |
| **METHODS** | | |  |
| Protocol and registration | 5 | Indicate if a review protocol exists, if and where it can be accessed (e.g., Web address), and, if available, provide registration information including registration number. | - |
| Eligibility criteria | 6 | Specify study characteristics (e.g., PICOS, length of follow-up) and report characteristics (e.g., years considered, language, publication status) used as criteria for eligibility, giving rationale. | 6-7 |
| **Section/topic** | **#** | **Checklist item** | **Reported on page #** |
| Information sources | 7 | Describe all information sources (e.g., databases with dates of coverage, contact with study authors to identify additional studies) in the search and date last searched. | 6-7 |
| Search | 8 | Present full electronic search strategy for at least one database, including any limits used, such that it could be repeated. | 6-7 |
| Study selection | 9 | State the process for selecting studies (i.e., screening, eligibility, included in systematic review, and, if applicable, included in the meta-analysis). | 6-7 |
| Data collection process | 10 | Describe method of data extraction from reports (e.g., piloted forms, independently, in duplicate) and any processes for obtaining and confirming data from investigators. | 7-8 |
| Data items | 11 | List and define all variables for which data were sought (e.g., PICOS, funding sources) and any assumptions and simplifications made. | 7-8 |
| Risk of bias in individual studies | 12 | Describe methods used for assessing risk of bias of individual studies (including specification of whether this was done at the study or outcome level), and how this information is to be used in any data synthesis. | 7 |
| Summary measures | 13 | State the principal summary measures (e.g., risk ratio, difference in means). | 7-8 |
| Synthesis of results | 14 | Describe the methods of handling data and combining results of studies, if done, including measures of consistency (e.g., I^2^) for each meta-analysis. | 7-8 |
| Risk of bias across studies | 15 | Specify any assessment of risk of bias that may affect the cumulative evidence (e.g., publication bias, selective reporting within studies). | 7 |
| Additional analyses | 16 | Describe methods of additional analyses (e.g., sensitivity or subgroup analyses, meta-regression), if done, indicating which were pre-specified. | 7 |
| Study selection | 17 | Give numbers of studies screened, assessed for eligibility, and included in the review, with reasons for exclusions at each stage, ideally with a flow diagram. | 8, Figure 1 |
| **Section/topic** | **#** | **Checklist item** | **Reported on page #** |
| Study characteristics | 18 | For each study, present characteristics for which data were extracted (e.g., study size, PICOS, follow-up period) and provide the citations. | 8,  Table 1 |
| Risk of bias within studies | 19 | Present data on risk of bias of each study and, if available, any outcome level assessment (see item 12). | 10-11,  Table S2 |
| Results of individual studies | 20 | For all outcomes considered (benefits or harms), present, for each study: (a) simple summary data for each intervention group (b) effect estimates and confidence intervals, ideally with a forest plot. | 10-11,  Table 1,  Figure S1-6 |
| Synthesis of results | 21 | Present results of each meta-analysis done, including confidence intervals and measures of consistency. | 9-11  Table 3 |
| Risk of bias across studies | 22 | Present results of any assessment of risk of bias across studies (see Item 15). | 10-11  Figure S7 |
| Additional analysis | 23 | Give results of additional analyses, if done (e.g., sensitivity or subgroup analyses, meta-regression [see Item 16]). | 11 |
| **DISCUSSION** |  |  |  |
| Summary of evidence | 24 | Summarize the main findings including the strength of evidence for each main outcome; consider their relevance to key groups (e.g., healthcare providers, users, and policy makers). | 11 |
| Limitations | 25 | Discuss limitations at study and outcome level (e.g., risk of bias), and at review-level (e.g., incomplete retrieval of identified research, reporting bias). | 15 |
| Conclusions | 26 | Provide a general interpretation of the results in the context of other evidence, and implications for future research. | 15-16 |
| **Section/topic** | **#** | **Checklist item** | **Reported on page #** |
| **FUNDING** |  |  |  |
| Funding | 27 | Describe sources of funding for the systematic review and other support (e.g., supply of data); role of funders for the systematic review. | 16 |
| Study selection | 17 | Give numbers of studies screened, assessed for eligibility, and included in the review, with reasons for exclusions at each stage, ideally with a flow diagram. | 8,  Figure 1 |
| Study characteristics | 18 | For each study, present characteristics for which data were extracted (e.g., study size, PICOS, follow-up period) and provide the citations. | 8,  Table 1 |

| **Table S2 PICOs, inclusion criteria and exclusion criteria of our meta-analysis during the database search** | | |
| --- | --- | --- |
| **PICOs** | **Inclusion criteria** | **Exclusion criteria** |
| **Population** | 1. Adults (over 18 years old). 2. Patients with COVID-19 were confirmed by real-time PCR 3. Severe cases with COVID-19: (1) Receiving treatment in intensive care unit. (2) Non-survivor. (3) SpO2<90% or required mechanical ventilation. (4) Complicated with ARDS and/or shock. (5) Hospitalized patients, compared with ambulatory patients. |  |
| **Intervention or exposure** | Exact cytokine level including interleukin-1 (IL-1), IL-6, IL-10, IL-18, and tumor necrosis factor. |  |
| **comparison** | Not required. |  |
| **Outcome** | No required. |  |
| **Study design** | 1. All articles were written in English. 2. All study designs including: case-control studies, cross-sectional studies, cohort studies and descriptive studies. 3. Year of Publication: December, 2019 to July 18, 2020. 4. Databases: PubMed, Embase, Web of Science. | 1. Review articles, editorials, comments, case reports, letters, and researches on pediatrics, pregnancy and obstetrics. 2. Articles in the MedRxiv or unaccepted articles. |

**Table S3 Risk of Bias of the included studies**

| **Score** | **A fair assessment of the end-point** | **The end-point adapted to the research goal** | **Sufficient numbers of patients** | **Sufficient data of interest** | **The definition of severe COVID-19** | **Including continuous patients** | **A clear purpose of the study** | **Author of included studies** |
| --- | --- | --- | --- | --- | --- | --- | --- | --- |
| 13 | 2 | 2 | 1 | 2 | 2 | 2 | 2 | Z Zhu et al (16) |
| 14 | 2 | 2 | 2 | 1 | 2 | 2 | 2 | Y Zheng et al (17) |
| 13 | 2 | 2 | 1 | 1 | 2 | 2 | 2 | C Zheng et al (18) |
| 14 | 2 | 2 | 2 | 1 | 2 | 2 | 2 | QH Zhang et al (19) |
| 13 | 2 | 2 | 1 | 2 | 2 | 2 | 2 | J Zhang et al (20) |
| 14 | 2 | 2 | 2 | 2 | 2 | 2 | 2 | D Zhang et al (21) |
| 13 | 2 | 2 | 2 | 1 | 2 | 2 | 2 | BC Zhang et al (22) |
| 14 | 2 | 2 | 2 | 2 | 2 | 2 | 2 | YL Yan et al (23) |
| 13 | 2 | 2 | 1 | 2 | 2 | 2 | 2 | B Xu et al (24) |
| 14 | 2 | 2 | 2 | 2 | 2 | 2 | 2 | YJ Wu et al (25) |
| 13 | 2 | 2 | 1 | 2 | 2 | 2 | 2 | Z Wang et al (26) |
| 13 | 2 | 2 | 1 | 2 | 2 | 2 | 2 | WJ Wang et al (27) |
| 13 | 2 | 2 | 1 | 2 | 2 | 2 | 2 | F Wang et al (28) |
| 13 | 2 | 2 | 1 | 2 | 2 | 2 | 2 | SX Wan et al (29) |
| 13 | 2 | 2 | 2 | 1 | 2 | 2 | 2 | A Vultaggio et al (30) |
| 14 | 2 | 2 | 2 | 2 | 2 | 2 | 2 | JB Tian et al (31) |
| 13 | 2 | 2 | 2 | 1 | 2 | 2 | 2 | H Sun et al (32) |
| 13 | 2 | 2 | 2 | 1 | 2 | 2 | 2 | JW Song, et al (33) |
| 12 | 2 | 2 | 1 | 1 | 2 | 2 | 2 | Q Luca et al (34) |
| 13 | 2 | 2 | 2 | 1 | 2 | 2 | 2 | M Austin R et al (35) |
| 13 | 2 | 2 | 2 | 1 | 2 | 2 | 2 | T Mikamiet al (36) |
| 14 | 2 | 2 | 2 | 2 | 2 | 2 | 2 | Y Liu et al (37) |
| 13 | 2 | 2 | 1 | 2 | 2 | 2 | 2 | SH Li et al (38) |
| 14 | 2 | 2 | 2 | 2 | 2 | 2 | 2 | H Huang et al (39) |
| 13 | 2 | 2 | 2 | 1 | 2 | 2 | 2 | HTobias et al (40) |
| 12 | 2 | 2 | 1 | 1 | 2 | 2 | 2 | Y Gao et al (41) |
| 14 | 2 | 2 | 2 | 2 | 2 | 2 | 2 | JH Gan et al (42) |
| 12 | 2 | 2 | 1 | 1 | 2 | 2 | 2 | D Michael et al (43) |
| 12 | 2 | 2 | 1 | 1 | 2 | 2 | 2 | D Erol et al (44) |
| 13 | 2 | 2 | 2 | 1 | 2 | 2 | 2 | X Chen et al (45) |
| 12 | 2 | 2 | 1 | 1 | 2 | 2 | 2 | G Chen et al (46) |
| 12 | 2 | 2 | 1 | 1 | 2 | 2 | 2 | CM Viviana et al (47) |
| 12 | 2 | 2 | 1 | 1 | 2 | 2 | 2 | E Burian et al (48) |

Figure S1. Forest Plot of meta-analysis of age in patients with severe COVID-19


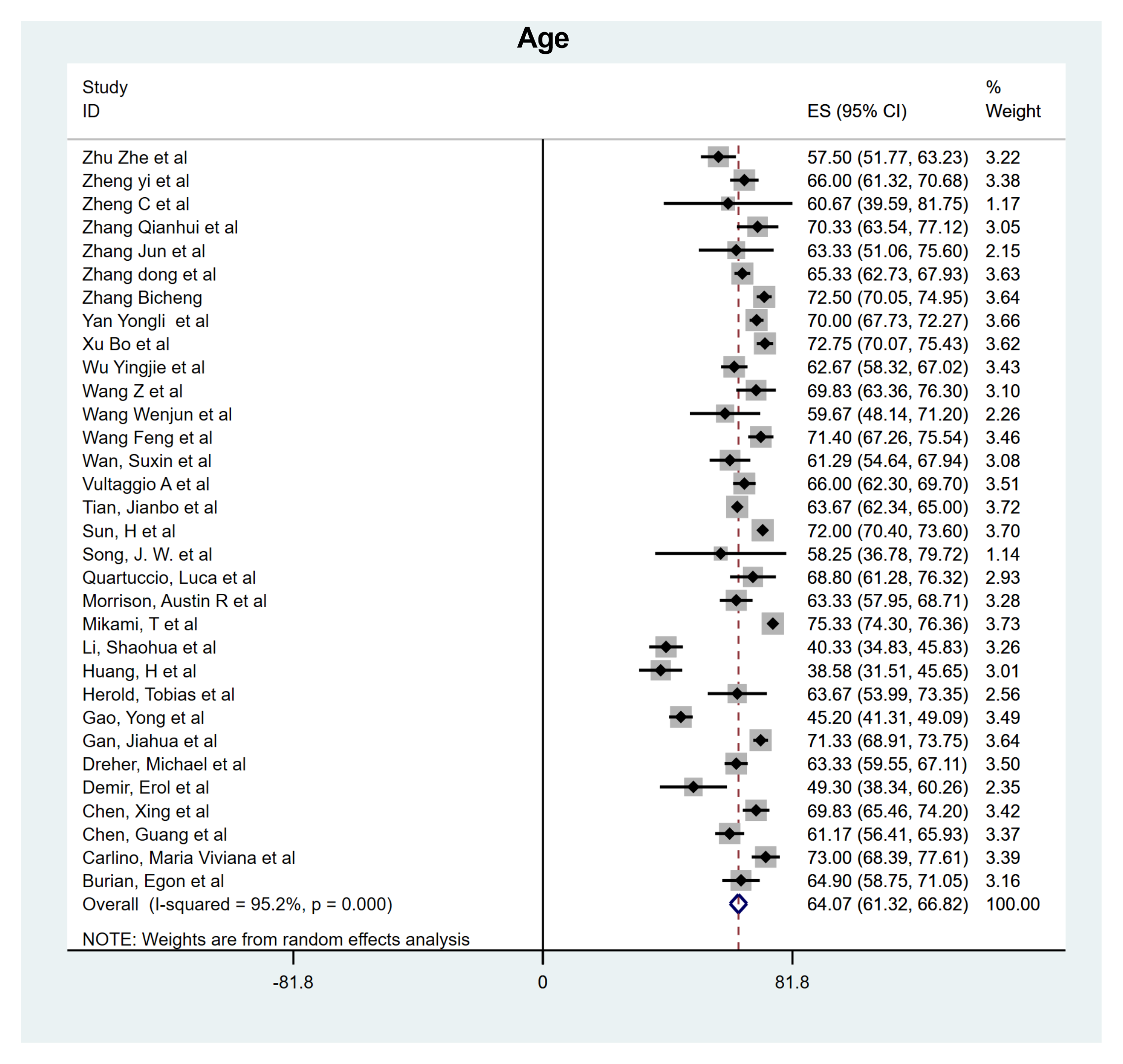


Figure S2. Forest Plot of meta-analysis of interleukin-1β in patients with severe COVID-19


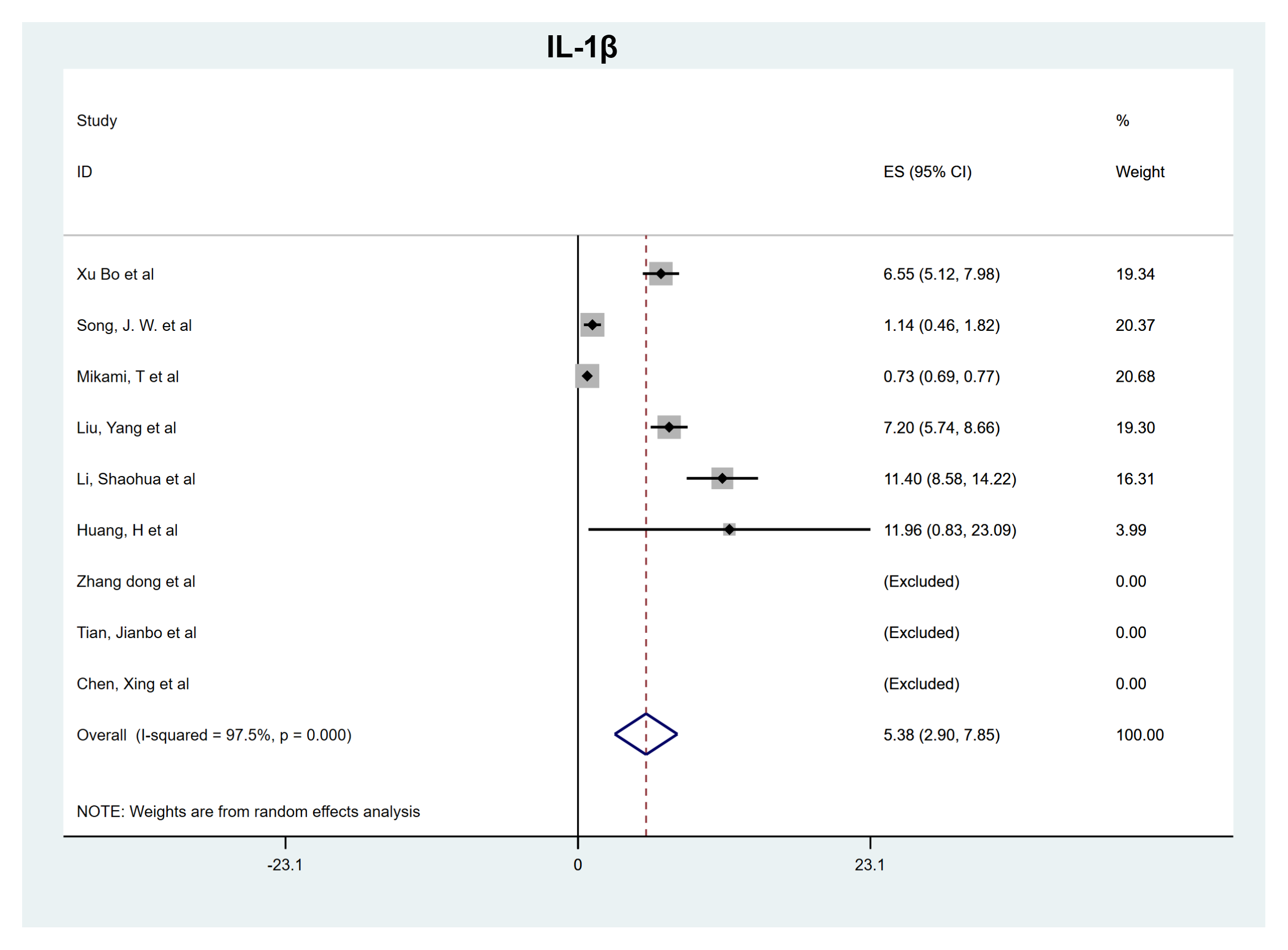


Figure S3. Forest Plot of meta-analysis of interleukin-6 in patients with severe COVID-19


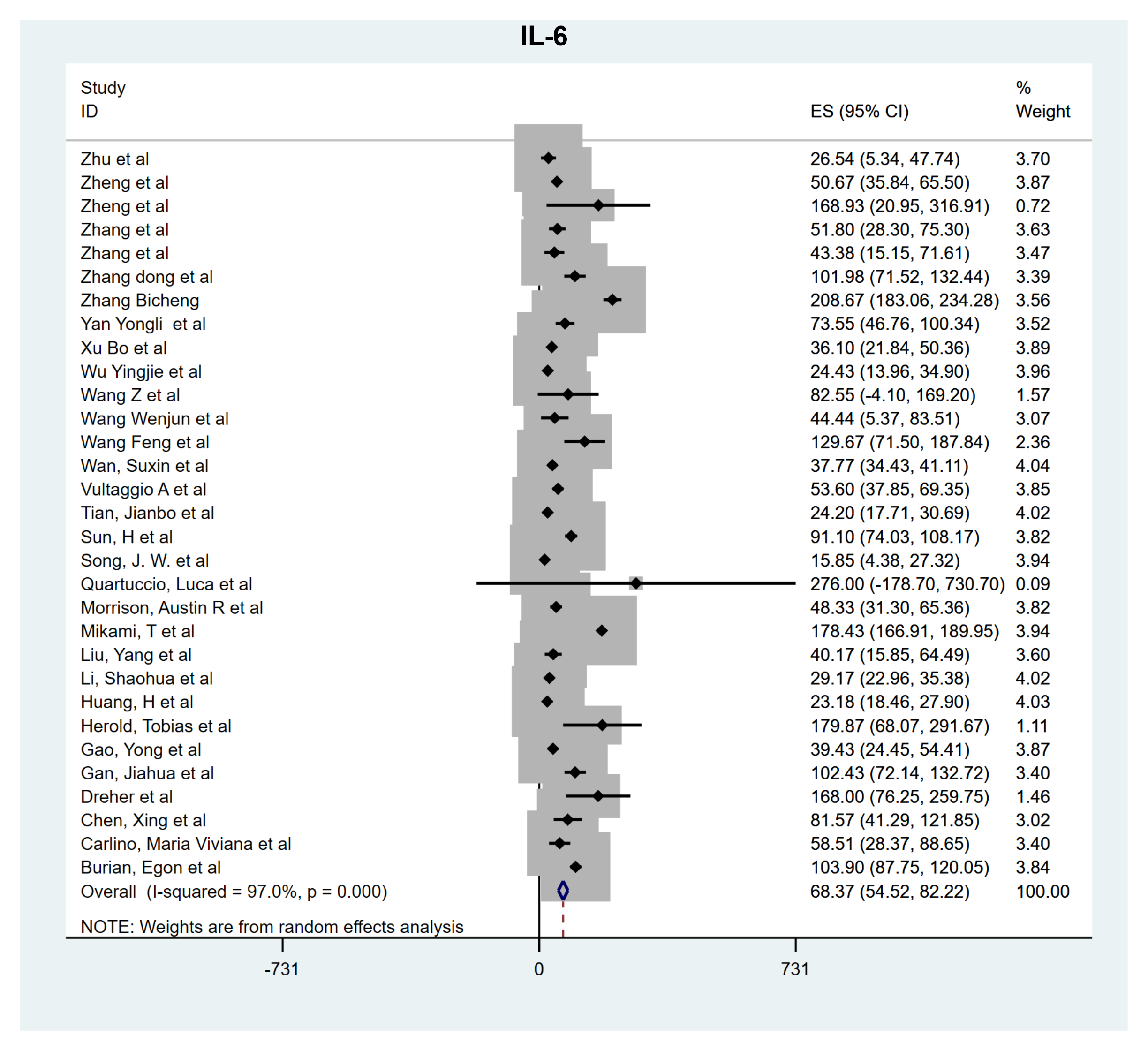


Figure S4. Forest Plot of meta-analysis of interleukin-10 in patients with severe COVID-19


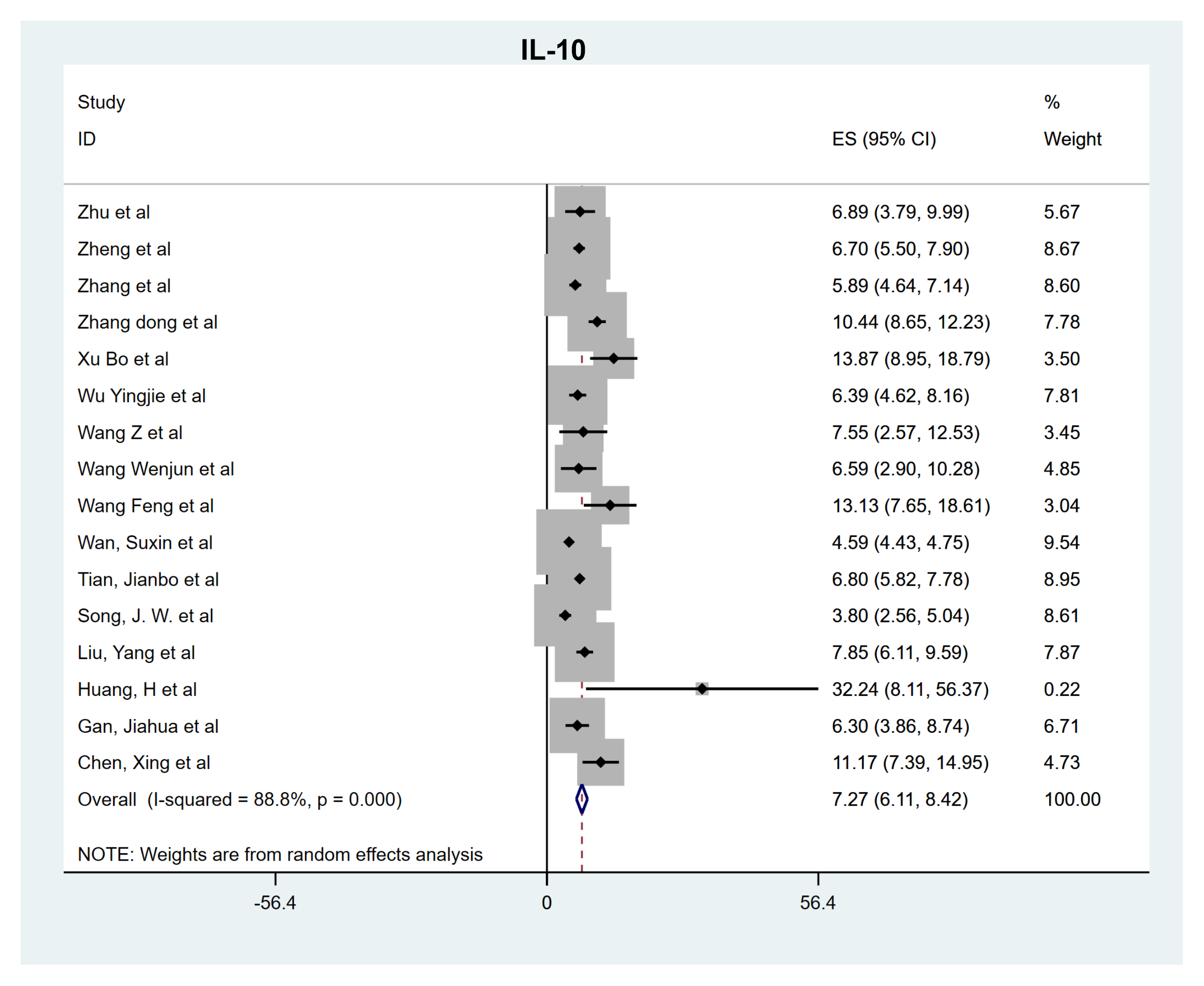


Figure S5. Forest Plot of meta-analysis of tumor necrosis factor-α in patients with severe COVID-19


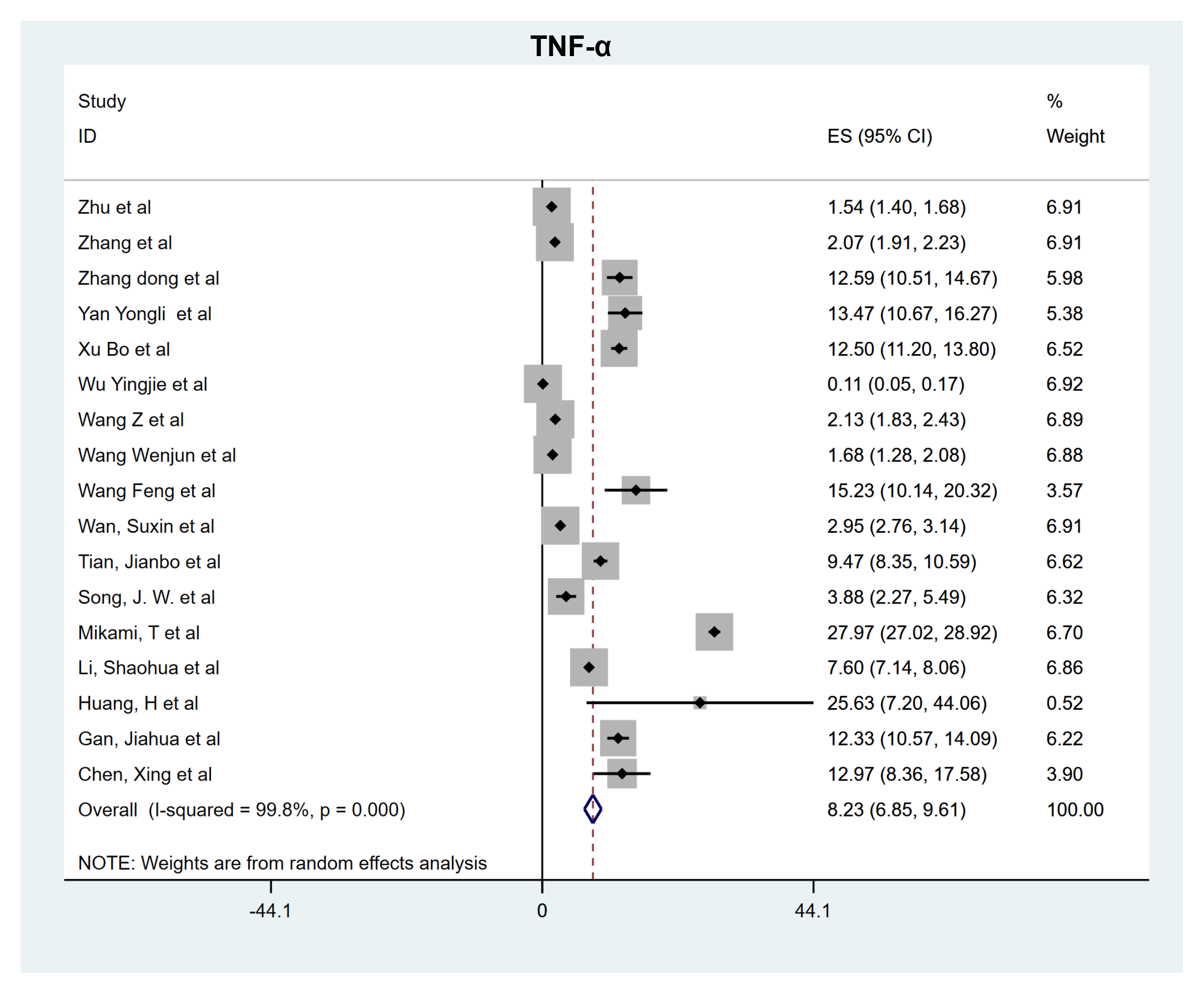


Figure S6. Forest Plot of meta-analysis of ferritin in patients with severe COVID-19


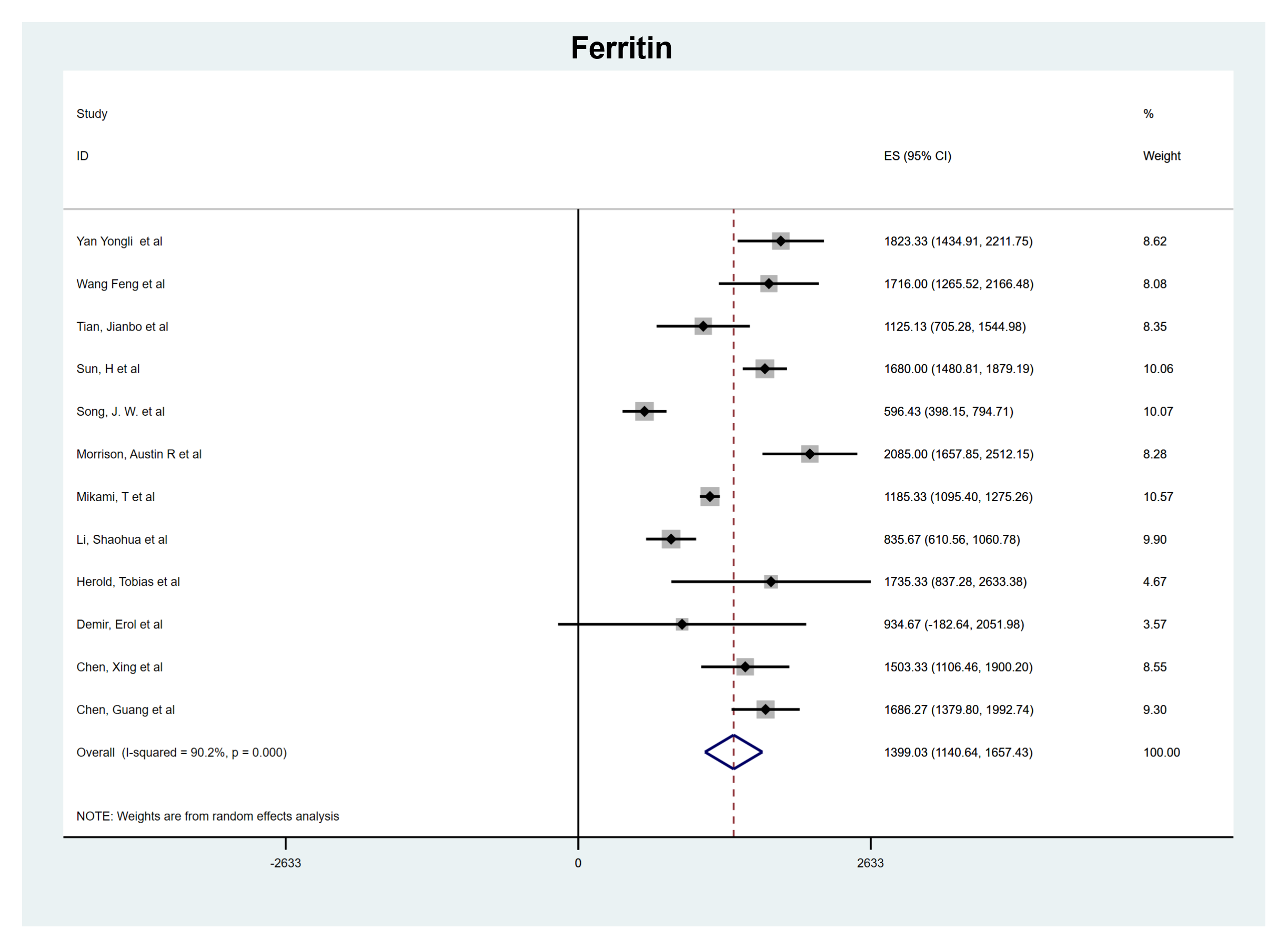


Figure S7. Funnel Plot of meta-analysis of ferritin in patients with severe COVID-19


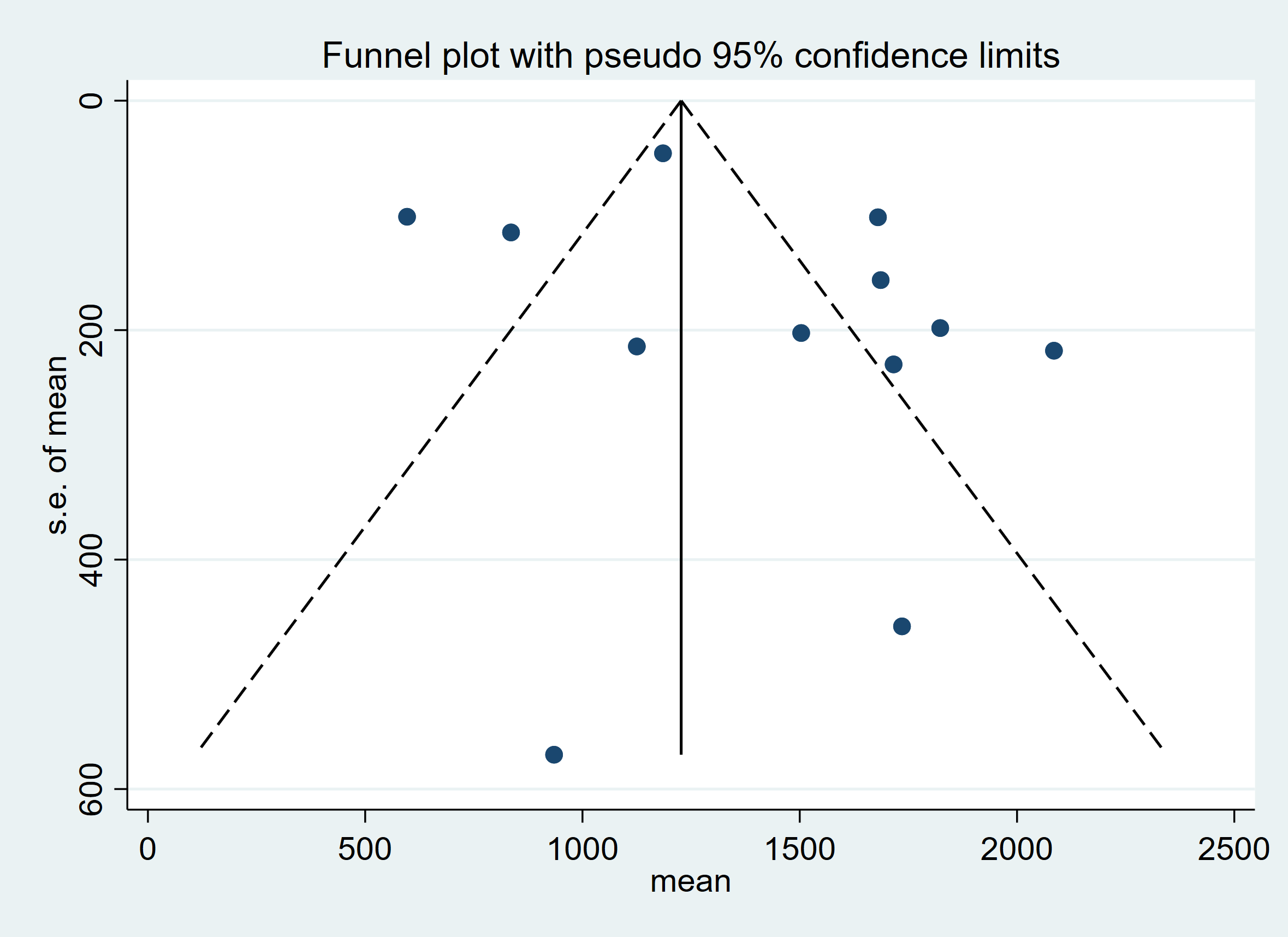


Figure S8. Funnel Plot of meta-analysis of age, IL-6, IL-10, and TNF-α in patients with severe COVID-19


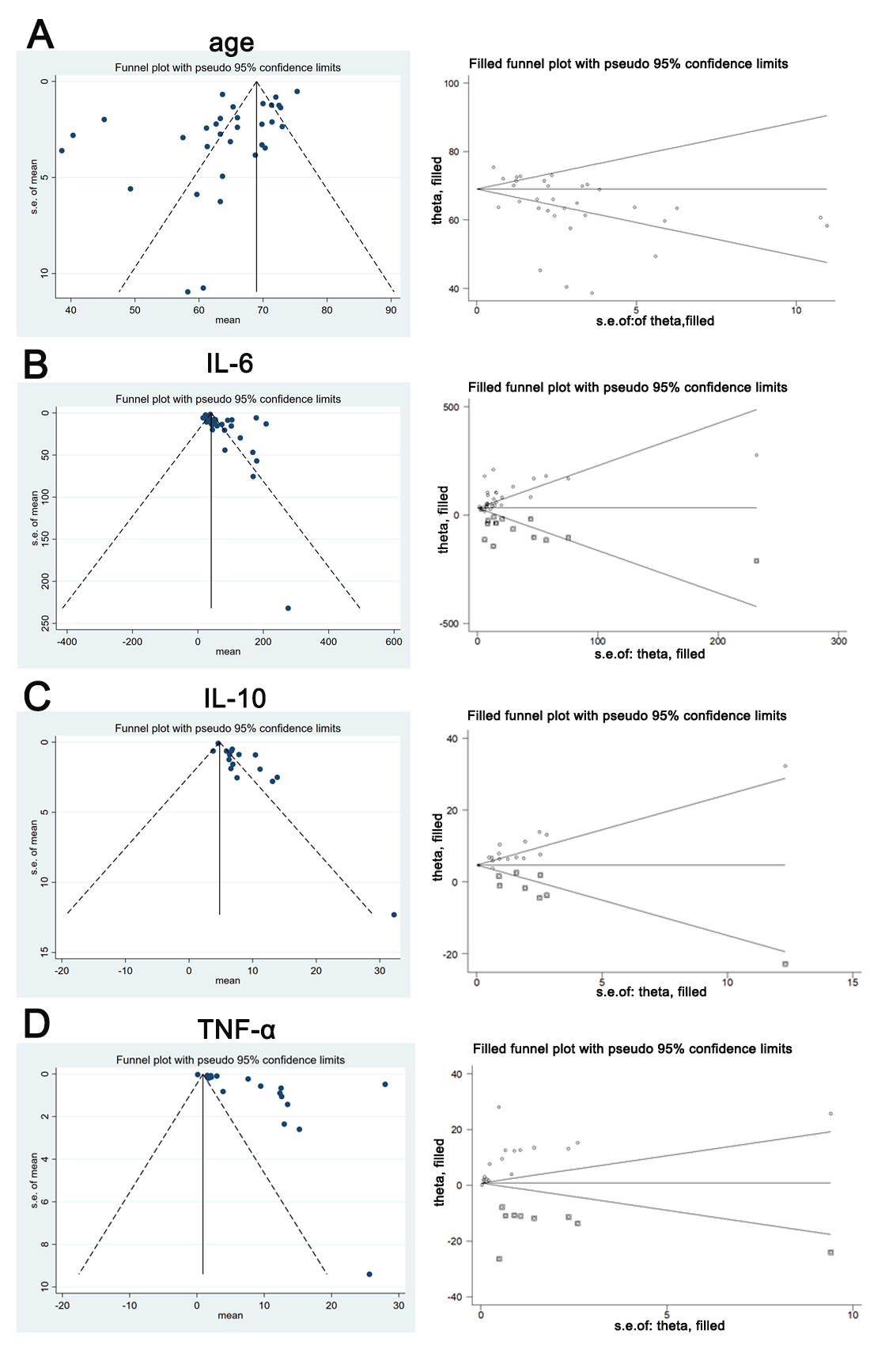


(A) Funnel Plot (left) and adjusted plot (right) in age. (B) Funnel Plot (left) and adjusted plot (right) in IL-6. (C) Funnel Plot (left) and adjusted plot (right) in IL-10. (D) Funnel Plot (left) and adjusted plot (right) in TNF-α.

**References**

16. Zhu Z, Cai T, Fan L, Lou K, Hua X, Huang Z, et al. Clinical value of immune-inflammatory parameters to assess the severity of coronavirus disease 2019. *International Journal of Infectious Diseases*. 95 (2020) 332-339. http://doi.org/10.1016/j.ijid.2020.04.041

17. Zheng Y, Sun L-j, Xu M, Pan J, Zhang Y-t, Fang X-l, et al. Clinical characteristics of 34 COVID-19 patients admitted to intensive care unit in Hangzhou, China. *Journal of Zhejiang University-Science B*. 21 (2020) 378-387. http://doi.org/10.1631/jzus.B2000174

18. Zheng C, Wang J, Guo H, Lu Z, Ma Y, Zhu Y, et al. Risk-adapted Treatment Strategy For COVID-19 Patients. *International Journal of Infectious Diseases*. 94 (2020) 74-77. http://doi.org/10.1016/j.ijid.2020.03.047

19. Zhang Q, Wei Y, Chen M, Wan Q, Chen X. Clinical analysis of risk factors for severe COVID-19 patients with type 2 diabetes. *Journal of diabetes and its complications*. (2020) 107666-107666. http://doi.org/10.1016/j.jdiacomp.2020.107666

20. Zhang J, Yu M, Tong S, Liu L-Y, Tang L-V. Predictive factors for disease progression in hospitalized patients with coronavirus disease 2019 in Wuhan, China. *Journal of Clinical Virology*. 127 (2020) http://doi.org/10.1016/j.jcv.2020.104392

21. Zhang D, Zhou X, Yan S, Tian R, Su L, Ding X, et al. Correlation between cytokines and coagulation-related parameters in patients with coronavirus disease 2019 admitted to ICU. *Clinica chimica acta; international journal of clinical chemistry*. (2020) http://doi.org/10.1016/j.cca.2020.07.002

22. Zhang B, Zhou X, Qiu Y, Song Y, Feng F, Feng J, et al. Clinical characteristics of 82 cases of death from COVID-19. *PloS one*. 15 (2020) e0235458-e0235458. http://doi.org/10.1371/journal.pone.0235458

23. Yan Y, Yang Y, Wang F, Ren H, Zhang S, Shi X, et al. Clinical characteristics and outcomes of patients with severe covid-19 with diabetes. *Bmj Open Diabetes Research & Care*. 8 (2020) http://doi.org/10.1136/bmjdrc-2020-001343

24. Xu B, Fan C-y, Wang A-l, Zou Y-l, Yu Y-h, He C, et al. Suppressed T cell-mediated immunity in patients with COVID-19: A clinical retrospective study in Wuhan, China. *Journal of Infection*. 81 (2020) E51-E60. http://doi.org/10.1016/j.jinf.2020.04.012

25. Wu Y, Huang X, Sun J, Xie T, Lei Y, Muhammad J, et al. Clinical Characteristics and Immune Injury Mechanisms in 71 Patients with COVID-19. *mSphere*. 5 (2020) http://doi.org/10.1128/mSphere.00362-20

26. Wang Z, Yang B, Li Q, Wen L, Zhang R. Clinical Features of 69 Cases With Coronavirus Disease 2019 in Wuhan, China. *Clin Infect Dis*. 71 (2020) 769-777. http://doi.org/10.1093/cid/ciaa272

27. Wang W, Liu X, Wu S, Chen S, Li Y, Nong L, et al. The Definition and Risks of Cytokine Release Syndrome in 11 COVID-19-Affected Critically Ill Patients with Pneumonia: Analysis of Disease Characteristics. *The Journal of infectious diseases*. (2020) http://doi.org/10.1093/infdis/jiaa387

28. Wang F, Yang Y, Dong K, Yan Y, Zhang S, Ren H, et al. CLINICAL CHARACTERISTICS OF 28 PATIENTS WITH DIABETES AND COVID-19 IN WUHAN, CHINA. *Endocrine Practice*. 26 (2020) 668-674. http://doi.org/10.4158/ep-2020-0108

29. Wan S, Yi Q, Fan S, Lv J, Zhang X, Guo L, et al. Relationships among lymphocyte subsets, cytokines, and the pulmonary inflammation index in coronavirus (COVID-19) infected patients. *British Journal of Haematology*. 189 (2020) 428-437. http://doi.org/10.1111/bjh.16659

30. Vultaggio A, Vivarelli E, Virgili G, Lucenteforte E, Bartoloni A, Nozzoli C, et al. Prompt Predicting of Early Clinical Deterioration of Moderate-to-Severe COVID-19 Patients: Usefulness of a Combined Score Using IL-6 in a Preliminary Study. *Journal of Allergy and Clinical Immunology: In Practice*. (2020) http://doi.org/10.1016/j.jaip.2020.06.013

31. Tian J, Yuan X, Xiao J, Zhong Q, Yang C, Liu B, et al. Clinical characteristics and risk factors associated with COVID-19 disease severity in patients with cancer in Wuhan, China: a multicentre, retrospective, cohort study. *Lancet Oncology*. 21 (2020) 893-903. http://doi.org/10.1016/s1470-2045(20)30309-0

32. Sun H, Ning R, Tao Y, Yu C, Deng X, Zhao C, et al. Risk Factors for Mortality in 244 Older Adults With COVID-19 in Wuhan, China: A Retrospective Study. *Journal of the American Geriatrics Society*. 68 (2020) E19-E23. http://doi.org/10.1111/jgs.16533

33. Song J W, Zhang C, Fan X, Meng F P, Xu Z, Xia P, et al. Immunological and inflammatory profiles in mild and severe cases of COVID-19. *Nat Commun*. 11 (2020) 3410. http://doi.org/10.1038/s41467-020-17240-2

34. Quartuccio L, Sonaglia A, Pecori D, Peghin M, Fabris M, Tascini C, et al. Higher levels of IL-6 early after tocilizumab distinguish survivors from non-survivors in COVID-19 pneumonia: a possible indication for deeper targeting IL-6. *Journal of medical virology*. (2020) http://doi.org/10.1002/jmv.26149

35. Morrison A R, Johnson J M, Griebe K M, Jones M C, Stine J J, Hencken L N, et al. Clinical characteristics and predictors of survival in adults with coronavirus disease 2019 receiving tocilizumab. *Journal of autoimmunity*. (2020) 102512-102512. http://doi.org/10.1016/j.jaut.2020.102512

36. Mikami T, Miyashita H, Yamada T, Harrington M, Steinberg D, Dunn A, et al. Risk Factors for Mortality in Patients with COVID-19 in New York City. *Journal of General Internal Medicine*. (2020) http://doi.org/10.1007/s11606-020-05983-z

37. Liu Y, Liao W, Wan L, Xiang T, Zhang W. Correlation Between Relative Nasopharyngeal Virus RNA Load and Lymphocyte Count Disease Severity in Patients with COVID-19. *Viral Immunology*. (2020) http://doi.org/10.1089/vim.2020.0062

38. Li S, Jiang L, Li X, Lin F, Wang Y, Li B, et al. Clinical and pathological investigation of patients with severe COVID-19. *JCI insight*. 5 (2020) http://doi.org/10.1172/jci.insight.138070

39. Huang H, Zhang M, Chen C, Zhang H, Wei Y, Tian J, et al. Clinical Characteristics of COVID-19 in patients with pre-existing ILD: A retrospective study in a single center in Wuhan, China. *Journal of Medical Virology*. (2020) http://doi.org/10.1002/jmv.26174

40. Herold T, Jurinovic V, Arnreich C, Lipworth B J, Hellmuth J C, Bergwelt-Baildon M v, et al. Elevated levels of IL-6 and CRP predict the need for mechanical ventilation in COVID-19. *The Journal of allergy and clinical immunology*. 146 (2020) 128-136.e124. http://doi.org/10.1016/j.jaci.2020.05.008

41. Gao Y, Li T, Han M, Li X, Wu D, Xu Y, et al. Diagnostic utility of clinical laboratory data determinations for patients with the severe COVID-19. *Journal of Medical Virology*. 92 (2020) 791-796. http://doi.org/10.1002/jmv.25770

42. Gan J, Li J, Li S, Yang C. Leucocyte Subsets Effectively Predict the Clinical Outcome of Patients With COVID-19 Pneumonia: A Retrospective Case-Control Study. *Frontiers in public health*. 8 (2020) 299-299. http://doi.org/10.3389/fpubh.2020.00299

43. Dreher M, Kersten A, Bickenbach J, Balfanz P, Hartmann B, Cornelissen C, et al. The Characteristics of 50 Hospitalized COVID-19 Patients With and Without ARDS. *Deutsches Arzteblatt International*. 117 (2020) 271-+. http://doi.org/10.3238/arztebl.2020.0271

44. Demir E, Uyar M, Parmaksiz E, Sinangil A, Yelken B, Dirim A B, et al. COVID-19 in kidney transplant recipients: A multicenter experience in Istanbul. *Transplant infectious disease : an official journal of the Transplantation Society*. (2020) e13371-e13371. http://doi.org/10.1111/tid.13371

45. Chen X, Yan L, Fei Y, Zhang C. Laboratory abnormalities and risk factors associated with in-hospital death in patients with severe COVID-19. *Journal of clinical laboratory analysis*. (2020) e23467-e23467. http://doi.org/10.1002/jcla.23467

46. Chen G, Wu D, Guo W, Cao Y, Huang D, Wang H, et al. Clinical and immunological features of severe and moderate coronavirus disease 2019. *The Journal of clinical investigation*. 130 (2020) 2620-2629. http://doi.org/10.1172/jci137244

47. Carlino M V, Valenti N, Cesaro F, Costanzo A, Cristiano G, Guarino M, et al. Predictors of Intensive Care Unit admission in patients with coronavirus disease 2019 (COVID-19). *Monaldi archives for chest disease = Archivio Monaldi per le malattie del torace*. 90 (2020) http://doi.org/10.4081/monaldi.2020.1410

48. Burian E, Jungmann F, Kaissis G A, Lohoefer F K, Spinner C D, Lahmer T, et al. Intensive Care Risk Estimation in COVID-19 Pneumonia Based on Clinical and Imaging Parameters: Experiences from the Munich Cohort. *Journal of Clinical Medicine*. 9 (2020) http://doi.org/10.3390/jcm9051514
